# Research on the Control Algorithm for a Brushless DC Motor Based on an Adaptive Extended Kalman Filter

**DOI:** 10.3390/s26031050

**Published:** 2026-02-05

**Authors:** Tong Jinwu, Zha Lifan, Lu Xinyun, Li Peng, Sun Jin, Liu Shujun

**Affiliations:** 1 Engineering Training Center, School of Applied Technology, Nanjing Institute of Technology, Nanjing 211167, China; 2 Nanjing Institute of Technology, Nanjing 211167, China; zhalifan1@163.com (Z.L.);; 3 School of Instrument Science and Engineering, Southeast University, Nanjing 210096, China; 4 Linko Semiconductor Co., Ltd., Nanjing 210096, China; 5 School of Internet of Things, Nanjing University of Posts and Telecommunications, Nanjing 210023, China

**Keywords:** brushless DC motor, sensorless control, extended Kalman filter, adaptive filtering, state estimation

## Abstract

**Highlights:**

**What are the main findings?**

- An Adaptive Extended Kalman Filter (AEKF) algorithm is proposed, featuring a robust weighting strategy and a dynamic forgetting factor to enable online innovation covariance updating.
- The designed AEKF demonstrates superior estimation accuracy, faster dynamic response, and enhanced robustness compared to the traditional EKF under various dynamic operating conditions.

**What are the implications of the main findings?**

- This work provides an effective and adaptive state estimation solution for high-performance sensorless control of BLDCs, directly addressing challenges from model uncertainties and external disturbances.
- The algorithm framework offers a valuable reference for the state observation of nonlinear electromechanical systems, contributing to the advancement of robust and intelligent control strategies in related fields.

**Abstract:**

To address the performance degradation of the traditional Extended Kalman Filter (EKF) in state estimation for sensorless brushless DC motor (BLDC) control under dynamic operating conditions, such as sudden speed and load changes—a degradation caused primarily by model mismatches—this paper proposes an Adaptive Extended Kalman Filter (AEKF) algorithm. The proposed algorithm incorporates a robust weighting strategy based on the Mahalanobis distance and a dynamically adjusted adaptive forgetting factor. This integration establishes an estimation mechanism capable of online updating of the innovation covariance, thereby enhancing the state observer’s adaptability to system uncertainties and external disturbances. Simulation results demonstrate that, compared to the traditional EKF, the designed AEKF algorithm significantly improves the estimation accuracy of rotor position and speed under various operating conditions, including low-speed start-up, speed step changes, and sudden load applications. Furthermore, it accelerates dynamic response, suppresses overshoot, and enhances the system’s disturbance rejection robustness. This work provides an effective state estimation solution for high-dynamic performance sensorless control of BLDC.

## 1. Introduction

Brushless DC motors (BLDCs) are widely used in numerous fields due to their advantages of simple structure, high power density, and strong reliability [1,2]. However, the high-performance operation of BLDCs traditionally depends on accurate real-time rotor position feedback, which is conventionally provided by physical sensors. During the operation of a BLDC, commutation typically requires real-time rotor position information obtained via position sensors. However, the use of such sensors not only increases system cost but also limits motor application in specific scenarios to some extent. Consequently, sensorless brushless DC motor control technology [3,4] has gradually become an important research direction in this field in recent years. Among related studies, mainstream sensorless control methods have been developed [5], including the back electromotive force (back-EMF) method [6], the inductance measurement method [7], the Model Reference Adaptive System (MRAS) technique [8], and state observer techniques [9,10].

Among back-EMF-based techniques, reference [11] proposes a back-EMF zero-crossing detection method, which indirectly obtains the zero-crossing points of the back-EMF in the non-conducting phase by measuring line voltages to determine rotor position. In inductance measurement techniques, reference [12] presents a method based on the inductance saturation effect, identifying commutation signals by injecting specific voltage vectors and comparing current responses. Furthermore, reference [13] suggests that injecting long and short voltage vectors with driving functionality can enhance dynamic performance while determining position. Although these methods are relatively simple in structure, their accuracy is susceptible to motor parameters and non-ideal operating conditions.

The Model Reference Adaptive System (MRAS) method, owing to its good adaptability and robustness, is widely used in control fields as it can effectively handle system parameter variations and external disturbances [14]. The core of this method lies in designing an adaptation law that enables the output of the controlled system to track that of a reference model, thereby maintaining good control performance during dynamic processes. In reference [15], an MRAS-based servo control strategy is proposed for automated guided vehicles, which improves control accuracy while effectively suppressing system oscillations. Reference [16] applies MRAS to field-oriented control, achieving accurate observation and regulation of motor speed and rotor position through a properly designed adaptation law.

State observer techniques represent another mainstream solution, among which the Sliding Mode Observer (SMO) is widely adopted due to its strong robustness. The SMO is constructed based on sliding mode control theory, typically using motor back-EMF and phase currents as input signals, and achieves motor state estimation by designing a sliding mode function. This method offers advantages such as fast response, relatively strong disturbance rejection capability, and lower dependency on model accuracy. However, its inherent nonlinear switching characteristic leads to chattering in the output. Therefore, the sliding mode gain and sliding surface require careful design to suppress chattering and enhance observation performance [17]. In related applications, reference [18] designed a high-speed SMO, achieving online identification of the motor rotor position and effectively avoiding the phase lag introduced by traditional low-pass filters. Reference [19] proposed an iterative SMO for estimating rotor position and speed, with experiments showing that this method improves dynamic response speed while reducing back-EMF estimation error. Traditional methods often combine the arctangent function to estimate rotor position and speed, but the inherent switching characteristic tends to introduce high-frequency chattering. To address this, some studies have employed Phase-Locked Loop (PLL) technology [20] for improvement and further enhanced the accuracy and stability of position tracking through normalization processing [21].

The Extended Kalman Filter (EKF), as an optimal state estimation algorithm, has also been used for dynamic tracking of rotor position. Initially proposed by the American scientist R.E. Kalman in the mid-20th century, this filter employs a recursive formulation [22], enabling simultaneous data acquisition and computation, thereby achieving real-time estimation of system states [23]. To apply it to nonlinear systems, the Extended Kalman Filter (EKF) was developed. The EKF demonstrates good performance in control effectiveness and estimation accuracy, exhibiting a certain degree of disturbance rejection capability. Even in the presence of measurement and system noise, it can provide a relatively accurate estimation for control systems [24]. With the advancement of power electronics technology, EKF has found widespread application in motor control, showing effectiveness in suppressing random disturbances and measurement noise, and possessing good adaptive regulation capability [25]. Reference [26] designed an EKF-based field-oriented control system for a three-phase induction motor, with simulations and experiments indicating that the system helps reduce torque ripple and speed oscillation. Reference [27] realized online identification of d-q axis inductance for automotive permanent magnet synchronous motors based on EKF, verifying the method’s effectiveness. Reference [28] proposed an adaptive EKF strategy for induction motors, with experiments demonstrating its ability to enhance system disturbance rejection and reliability.

However, several limitations persist in the application of EKF to motor control. Firstly, due to the need for online calculation of the Jacobian matrix and execution of complex matrix operations, its computational burden is significant, resulting in weak real-time performance. This makes it difficult to meet control cycle requirements, especially under high-speed or highly dynamic operating conditions [29]. Secondly, EKF is sensitive to model errors and noise statistics. Its estimation accuracy and disturbance rejection capability degrade under strong disturbances or significant parameter variations, easily leading to performance deterioration. Furthermore, during low-speed motor operation or when back-EMF is weak, the signal-to-noise ratio of the observed signal decreases. This can cause substantial deviations in EKF’s estimation of rotor position [30], potentially leading to observer failure and affecting low-speed performance and system stability. Therefore, although EKF has achieved certain success in motor control, the aforementioned issues still constrain its application in higher-performance scenarios [31], necessitating algorithm improvements to enhance system performance.

To address the problems of decreased estimation accuracy and response lag in sensorless brushless DC motor (BLDC) control using traditional EKF—problems stemming from model mismatch, uncertain noise statistics, and insufficient adaptability to dynamic operating conditions—this paper designs an Adaptive Extended Kalman Filter (AEKF) algorithm. Building upon the EKF framework, the proposed algorithm introduces a robust weighting strategy based on Mahalanobis distance and a dynamically adjusted adaptive forgetting factor. This constructs an adaptive estimation mechanism capable of online updating of the innovation covariance, thereby significantly enhancing the state observer’s robustness to system parameter variations and external disturbances. This research focuses on the design of this algorithm. It first establishes the state-space model of the sensorless BLDC system and elaborates on the implementation steps of the traditional EKF. Subsequently, it details the improvement strategies and implementation process of the proposed Adaptive Extended Kalman Filter (AEKF). Finally, the superior performance of the AEKF is verified through simulation experiments under various operating conditions, including low-speed start-up, speed step changes, and sudden load applications. This work aims to provide a more reliable state estimation solution for high-dynamic sensorless control systems of BLDCs.

## 2. Modeling of Sensorless BLDC System and Establishment of EKF State Estimation

### 2.1. BLDC System Modeling

The brushless DC motor (BLDC) is widely used in servo drives, electric vehicles, and aerospace applications due to its simple structure, high efficiency, and ease of control. A key characteristic of the BLDC is its trapezoidal back electromotive force (back-EMF) waveform. This characteristic makes its control and modeling more challenging, especially in sensorless control scenarios, where the accuracy of rotor position estimation directly determines the motor’s operational performance.

This paper considers the BLDC as the control plant. Neglecting effects such as iron losses and magnetic saturation, the voltage equations for its three-phase windings are as follows:

(1) $$\left\{\begin{array}{rr} u_{a}(t) & =R_{s}i_{a}(t)+L_{s}\frac{di_{a}(t)}{\mathrm{dt}}+e_{a}(t),\\ u_{b}(t) & =R_{s}i_{b}(t)+L_{s}\frac{di_{b}(t)}{\mathrm{dt}}+e_{b}(t),\\ u_{c}(t) & =R_{s}i_{c}(t)+L_{s}\frac{di_{c}(t)}{\mathrm{dt}}+e_{c}(t). \end{array}\right.$$

In the equations, $u_{a}$, $u_{b}$, $u_{c}$ represent the three-phase stator voltages; $i_{a}$, $i_{b}$, $i_{c}$ represent the three-phase stator currents; $R_{s}$ is the stator resistance; $L_{s}$ is the stator inductance; $e_{a}$, $e_{b}$, $e_{c}$ are the back electromotive forces (back-EMF).

The relationship between the back electromotive force and the electrical angular velocity $\omega_{e}$ as well as the rotor position angle θ can be expressed as follows:

(2) $$e_{\mathrm{abc}}=K_{e}\omega_{e}f(\theta ).$$

In the equations: $K_{e}$ is the back-EMF constant; f(θ) is the normalized trapezoidal function.

To transform the originally complex three-phase AC problem into a relatively simpler problem in a two-phase stationary coordinate system, the Clarke transformation is employed to map the three-phase quantities onto the αβ coordinate system:

(3) $$\left[\begin{array}{c} i_{\alpha }\\ i_{\beta } \end{array}\right]=\frac{2}{3}\left[\begin{array}{ccc} 1 & -\frac{1}{2} & -\frac{1}{2}\\ 0 & \frac{\sqrt{3}}{2} & -\frac{\sqrt{3}}{2} \end{array}\right]\left[\begin{array}{c} i_{a}\\ i_{b}\\ i_{c} \end{array}\right].$$

The relationship between the rate of change in current, voltage, and back-EMF can be expressed as follows:

(4) $$\left\{\begin{array}{rr} \frac{di_{\alpha }}{\mathrm{dt}} & =-\frac{R_{s}}{L_{s}}i_{\alpha }+\frac{1}{L_{s}}(u_{\alpha }-e_{\alpha }),\\ \frac{di_{\beta }}{\mathrm{dt}} & =-\frac{R_{s}}{L_{s}}i_{\beta }+\frac{1}{L_{s}}{(u}_{\beta }-e_{\beta }). \end{array}\right.$$

The electromagnetic torque equation can be expressed as follows:

(5) $$T_{e}=\frac{3P(e_{\alpha }i_{\alpha }+e_{\beta }i_{\beta })}{4\omega_{e}}.$$

The rotor motion equation is given as follows:

(6) $$J\frac{d\omega_{e}}{\mathrm{dt}}=T_{e}-T_{L}-B\omega_{e}.$$

In the equations: P  is the number of pole pairs; J  is the moment of inertia; $T_{L}$ is the load torque; B is the friction coefficient.

At this point, the state variables can be defined as follows:

(7) $${x=\left[i_{\alpha },i_{\beta },\omega_{e},\theta \right]}^{T}.$$

The nonlinear function of the system is as follows:

(8) $$\;f(x,u)=\left[\begin{array}{c} -\frac{R_{s}}{L_{s}}i_{\alpha }+\frac{1}{L_{s}}(u_{\alpha }-e_{\alpha }(\theta ,\omega_{e}))\\ -\frac{R_{s}}{L_{s}}i_{\beta }+\frac{1}{L_{s}}(u_{\beta }-e_{\beta }(\theta ,\omega_{e}))\\ \frac{1}{J}(\frac{3P(e_{\alpha }i_{\alpha }+e_{\beta }i_{\beta })}{4\omega_{e}}-T_{L}-B\omega_{e})\\ \omega_{e} \end{array}\right].$$

Combining the above equations, the state equation and the output equation of the BLDC system are established as follows:

(9) $$\left\{\begin{array}{l} \dot{x}=f(x,u)+w,\\ y=\mathrm{Hx}+v. \end{array}\right.$$

In the equation, the input vector is $\;u\;=\;{\left[u_{\alpha },u_{\beta }\right]}^{T}$; the observation function is $\;H\;=\;{\left[i_{\alpha },i_{\beta }\right]}^{T}$; w is the process noise and v is the measurement noise, both assumed to be zero-mean Gaussian white noises.

### 2.2. Formulation of EKF State Estimation

The Extended Kalman Filter (EKF) is an extension of the Kalman Filter for nonlinear systems. Its fundamental principle involves performing local linearization of the nonlinear system using Taylor series expansion and achieving optimal estimation of the state variables through iterative optimization. By applying linearization and discretization to the BLDC system and combining it with the above equations, the Extended Kalman state equation is established as follows:

(10) $$\left\{\begin{array}{l} x_{k}=f(x_{k},u_{k})+w_{k},\\ y_{k}=h(x_{k})+v_{k}. \end{array}\right.$$

In the equation, the process noise is $w_{k}\in N(0,Q)$; the measurement noise is $v_{k}\in N(0,R)$; f is the nonlinear state equation obtained by discretizing Equations $\left\{\begin{array}{rr} \frac{di_{\alpha }}{\mathrm{dt}} & =-\frac{R_{s}}{L_{s}}i_{\alpha }+\frac{1}{L_{s}}(u_{\alpha }-e_{\alpha }),\\ \frac{di_{\beta }}{\mathrm{dt}} & =-\frac{R_{s}}{L_{s}}i_{\beta }+\frac{1}{L_{s}}{(u}_{\beta }-e_{\beta }). \end{array}\right.$ (4) $J\frac{d\omega_{e}}{\mathrm{dt}}=T_{e}{-T}_{L}-B\omega_{e}\;$(6); h is the measurement function.

The filtering procedure of the EKF is as follows:

(1) The state prediction is as follows:
  (11) $${\hat{x}}_{k|k-1}=f({\hat{x}}_{k-1|k-1},u_{k-1}).$$
(2) The covariance prediction is as follows:
  (12) $$P_{k|k-1}=F_{k}P_{k-1|k-1}F_{k}^{T}+Q.$$

In the equations: $F_{k}=\left.\frac{\partial y}{\partial x}\right|{\hat{x}}_{k-1}$ is the Jacobian matrix.

(3) Kalman gain calculation:
  (13) $$K_{k}=P_{k|k-1}H_{k}^{T}(H_{k}P_{k|k-1}H_{k}^{T}+R{)}^{-1}.$$

In the equations: $H_{k}=\frac{\partial h}{\partial x}.$

(4) The state update is as follows:
  (14) $${\hat{x}}_{k|k}={\hat{x}}_{k|k-1}+K_{k}(y_{k}-h({\hat{x}}_{k|k-1}))\;.$$
(5) The covariance update is as follows:
  (15) $$P_{k|k}=(I-K_{k}H_{k})P_{k|k-1}$$

However, the practical application of the Extended Kalman Filter in the field of motor control still faces several challenges. Firstly, due to the need for online calculation of the Jacobian matrix and execution of complex matrix operations, its significant computational burden limits real-time performance under high-speed or high-dynamic operating conditions, making it difficult to meet the requirements of short control cycles. Secondly, the EKF is sensitive to model errors and noise statistics; its estimation accuracy and disturbance rejection capability are prone to degradation under strong disturbances or significant parameter variations, which can even lead to performance deterioration. Furthermore, during low-speed motor operation or when the back-EMF amplitude is low, the signal-to-noise ratio of the observed signal decreases, which can easily cause large deviations or even failure in rotor position estimation, thereby affecting the low-speed stability and overall performance of the system. Although the EKF has achieved certain success in motor state estimation, the aforementioned limitations still constrain its application in scenarios requiring higher dynamics and a wider operating range.

Based on this, this paper aims to design an Adaptive Extended Kalman Filter (AEKF) algorithm. By enhancing its online identification and adaptive adjustment capabilities regarding innovation statistics, the proposed algorithm seeks to improve the robustness, adaptability, and estimation accuracy of the state observer under model uncertainties and external disturbances. This work is intended to advance the development of sensorless motor control systems towards applications demanding higher dynamics and a broader operating range.

## 3. AEKF Improvement Strategy Based on Adaptive Innovation

Unlike classical adaptive filters that employ a single mechanism to handle all uncertainties, the proposed AEKF introduces a differentiated uncertainty-handling strategy by online diagnosis of the innovation sequence. The Mahalanobis distance of the innovation serves as a real-time discriminator: robust weighting suppresses sporadic, large-magnitude outliers (impulsive uncertainties), while a dynamic forgetting factor smoothly adjusts the reliance on historical data to track persistent statistical drift. This composite adaptive mechanism, which classifies uncertainties and applies distinct countermeasures, constitutes the core theoretical distinction of the proposed method.

### 3.1. Innovation Covariance Estimation

To ensure that the innovation covariance reflects recent observation statistics while remaining robust to outliers, we adopt a weighted, regularized exponential recursion form and adaptively adjust the weights based on a robust function and the Mahalanobis distance.

The innovation is defined as follows:

(16) $$\varepsilon_{k}=y_{k}-h({\hat{x}}_{k|k-1})\;.$$

In the equation, $\varepsilon_{k}\in R^{m}$; $y_{k}$ represents the measured quantities $i_{\alpha }$ and $i_{\beta }$ of the BLDC.

The Mahalanobis distance is defined as follows:

(17) $$d_{k}=\varepsilon_{k}^{T}{\left({\hat{C}}_{k-1}+\delta I\right)}^{-1}\varepsilon_{k}.$$

In the equation, ${\hat{C}}_{k-1}$ is the innovation covariance estimated at the previous time step; δ > 0 is a numerical regularization term to prevent matrix singularity; a larger $d_{k}$ indicates more anomalous current statistics. This formula is used for the dynamic adjustment of the forgetting factor and robust weighting.

Robust weighting:

(18) $$w_{k}=\Phi (d_{k})=\left\{\begin{array}{ll} 1, & d_{k}\leq \tau \\ \frac{\tau }{d_{k}}, & d_{k}>\tau \end{array}\right.$$

In the equation, τ is the threshold (taken as the 95th percentile of the chi-square distribution).

Adaptive forgetting factor λ(k) adjustment rule:

(19) $$\lambda_{k}=\lambda_{\infty }+(\lambda_{0}+\lambda_{\infty })e^{-\eta d_{k}}.$$

In the equations: $\lambda_{0}$ is the initial value of 0.98; $\lambda_{\infty }$ is the minimum value of 0.6. This formulation actively increases the focus on the current innovation when a sudden change occurs, while maintaining smoothness when the system is in a steady state.

Robust exponentially weighted innovation covariance recursion:

(20) $${\hat{C}}_{k}=\lambda_{k}{\hat{C}}_{k-1}+(1-\lambda_{k})w_{k}{\varepsilon_{k}\varepsilon }_{k}^{T}+\epsilon I.$$

In the equations: ${\hat{C}}_{k}\in R^{m\times m}$, with ${\hat{C}}_{k}$ being the estimated innovation covariance. This formulation balances recent statistics with robustness, enabling a rapid response to abrupt changes while suppressing the impact of isolated errors on the statistics.

### 3.2. Adaptive Kalman Gain and Robust Update

Combining the above equations, along with the state update, a new state prediction equation is established for the system as follows:

(21) $${\hat{x}}_{k|k-1}={\hat{x}}_{k-1|k-1}+T_{s}f({\hat{x}}_{k-1|k-1},u_{k-1}).$$

In the equation, $T_{s}$ is the sampling period; this formula utilizes the forward Euler approximation to discretize the continuous-time state evolution process, thereby enabling the prediction of the state at time k based on the state at time k−1.

State Jacobian matrix:

(22) $$F_{k}={\left.\frac{\partial f(x,u)}{\partial x}\right|}_{x={\hat{x}}_{k-1|k-1},u=u_{k-1}}.$$

In the equation, the Jacobian matrix $F_{k}$ is the first-order partial derivative matrix of the state transition function f(x,u) with respect to the state vector x.

Covariance Prediction:

(23) $$P_{k|k-1}=F_{k}P_{k-1|k-1}F_{k}^{T}+Q_{k}.$$

In the equation, the $Q_{k}$ matrix enables the filter to better adapt to dynamic changes in the system.

The observation Jacobian matrix is as follows:

(24) $$H_{k}={\left.\frac{\partial h(x)}{\partial x}\right|}_{x={\hat{x}}_{k|k-1}}.$$

In the equation, h(x) is the observation function; $H_{k}$ is the observation Jacobian matrix, which linearizes the nonlinear observation function h(x).

After completing the calculations for the state Jacobian matrix, covariance prediction, and observation Jacobian matrix, the next step is to perform the gain calculation, state update, and covariance update for the incremental adaptive Kalman filter.

The calculation of the adaptive Kalman gain is as follows:

(25) $$K_{k}=P_{k|k-1}H_{k}^{T}{\hat{C}}_{k}^{-1}.$$

In the equation, $K_{k}$ is the adaptive Kalman gain matrix; $H_{k}^{T}$ is the transpose of the observation Jacobian matrix; this equation provides the correction gain for the subsequent state update.

State Update:

(26) $${\hat{x}}_{k|k}={\hat{x}}_{k|k-1}+K_{k}\varepsilon_{k}.$$

This equation combines the adaptive Kalman gain with the observation residual to correct the predicted state, yielding an optimal estimate that is closer to the true state.

Covariance Update:

(27) $$P_{k|k}=(I-K_{k}H_{k})P_{k|k-1}{\left(I-K_{k}H_{k}\right)}^{T}+K_{k}{\hat{C}}_{k}K_{k}^{T}.$$

In the equation, I is the identity matrix; this equation updates the covariance matrix of the state estimate. This form ensures the positive definiteness and symmetry of the covariance matrix and exhibits superior numerical properties compared to the traditional Extended Kalman Filter.

### 3.3. Algorithm Implementation Flowchart

Given that the proposed Adaptive Extended Kalman Filter (AEKF) algorithm integrates multiple modules, including robust weighting adjustment, adaptive forgetting factor update, and online innovation covariance estimation, its recursive structure possesses a certain level of complexity. To clearly present the logical connections and execution sequence of each step in the algorithm, this paper summarizes the overall implementation flow of the AEKF in the form of a flowchart.

**Figure 1:**
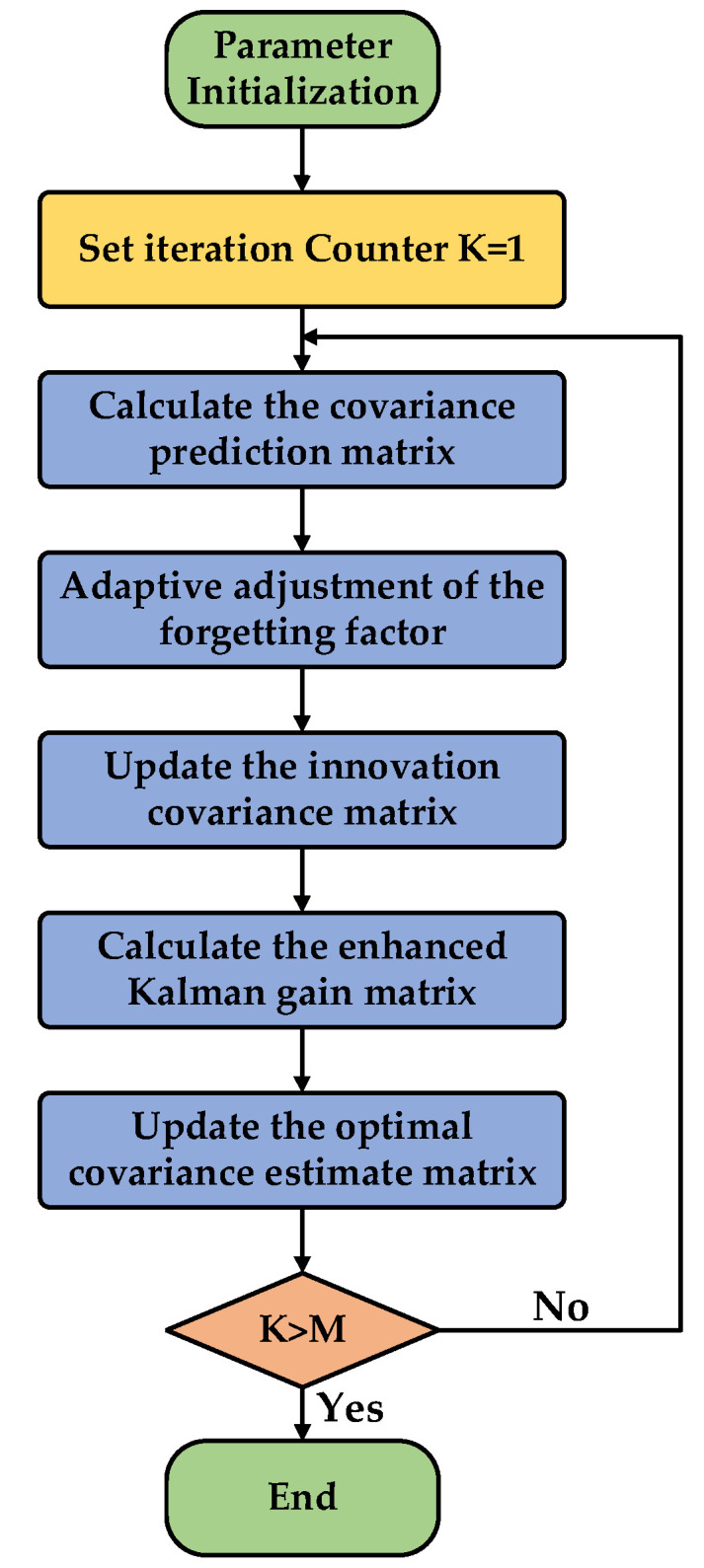
AEKF algorithm flowchart.

The complete implementation flow of the Adaptive Extended Kalman Filter (AEKF) algorithm proposed in this paper is illustrated in Figure 1, with the specific steps described as follows:

Step 1: Initialization

Set the initial state estimate ${\hat{x}}_{0|0}$, error covariance matrix $P_{0|0}$, process noise covariance matrix $Q_{0}$, measurement noise covariance matrix $R_{0}$, and initial innovation covariance estimate ${\hat{C}}_{0}$. Simultaneously, configure the parameters for the adaptive forgetting factor: $\lambda_{0}$, $\lambda_{\infty }$, η, and the robust weighting threshold τ.

Step 2: State Prediction

Based on the optimal state estimate ${\hat{x}}_{k-1|k-1}$ and the input $u_{k-1}$ at time k−1, perform a one-step state prediction using the discretized system state equation (Equation ${\hat{x}}_{k|k-1}={\hat{x}}_{k-1|k-1}+T_{s}f({\hat{x}}_{k-1|k-1},u_{k-1})\;$ (21)) to obtain the predicted state ${\hat{x}}_{k|k-1}$ at time k.

Step 3: Covariance Prediction

Calculate the state transition Jacobian matrix $F_{k}$ (Equation $F_{k}={\left.\frac{\partial f(x,u)}{\partial x}\right|}_{x={\hat{x}}_{k-1|k-1},u=u_{k-1}}\;$(22)). Then, compute the predicted error covariance matrix $P_{k|k-1}$ according to the following equation: $P_{k|k-1}=F_{k}P_{k-1|k-1}F_{k}^{T}+Q_{k}$ (23).

Step 4: Innovation Calculation and Mahalanobis Distance Assessment

Obtain the actual measurement $y_{k}$ at time k, and calculate the innovation $\varepsilon_{k}$ according to the following equation: $\varepsilon_{k}=y_{k}-h({\hat{x}}_{k|k-1})\;\;$ (16). Next, compute the Mahalanobis distance $d_{k}$ of the current innovation based on the following equation: $d_{k}=\varepsilon_{k}^{T}{\left({\hat{C}}_{k-1}+\delta I\right)}^{-1}\varepsilon_{k}$ (17).

Step 5: Adaptive Parameter Adjustment

Based on the calculated Mahalanobis distance $d_{k}$:

(1) Calculate the robust weight $w_{k}$ according to the following equation: $w_{k}=\Phi (d_{k})=\left\{\begin{array}{ll} 1, & d_{k}\leq \tau \\ \frac{\tau }{d_{k}}, & d_{k}>\tau \end{array}\right.$(18). If $d_{k}\leq \tau$, the current statistics are considered normal; if $d_{k\;}>\;\tau$, the influence of outliers is mitigated.
(2) The adaptive forgetting factor $\lambda_{k}$ is dynamically adjusted according to the Equation Adaptive forgetting factor λ(k) adjustment rule: $\lambda_{k}=\lambda_{\infty }+(\lambda_{0}+\lambda_{\infty })e^{-\eta d_{k}}$ (19). When a sudden system change occurs (i.e., $d_{k}$ increases), $\lambda_{k}$ decreases, directing the algorithm’s focus more towards the current innovation; when the system is in a steady state, $\lambda_{k}$ increases to maintain smooth filtering.

Step 6: Innovation Covariance Online Estimation

Using the adjusted $\lambda_{k}$ and $w_{k}$, recursively update the innovation covariance estimate ${\hat{C}}_{k}$ according to the following equation: ${\hat{C}}_{k}=\lambda_{k}{\hat{C}}_{k-1}+(1-\lambda_{k})w_{k}{\varepsilon_{k}\varepsilon }_{k}^{T}+$*ϵ*I (20). This step enables the algorithm to dynamically reflect the latest observational statistics of the system and enhances its robustness against outliers.

Step 7: Calculation of Observation Jacobian Matrix and Adaptive Kalman Gain

Compute the observation Jacobian matrix $H_{k}$ (Equation $H_{k}={\left.\frac{\partial h(x)}{\partial x}\right|}_{x={\hat{x}}_{k|k-1}}\;$ (24)). Then, utilizing the updated ${\hat{C}}_{k}$, calculate the adaptive Kalman gain matrix $K_{k}$ according to the following equation: $K_{k}=P_{k|k-1}H_{k}^{T}{\hat{C}}_{k}^{-1}$ (25).

Step 8: State Update and Covariance Update

(1) State Update: Calculate the optimal state estimate ${\hat{x}}_{k|k}$ at time k according to the following equation: ${\hat{x}}_{k|k}={\hat{x}}_{k|k-1}+K_{k}\varepsilon_{k}$ (26), by combining the state prediction ${\hat{x}}_{k|k-1}$, the Kalman gain $K_{k}$, and the innovation $\varepsilon_{k}$.
(2) Covariance Update: Update the error covariance matrix $P_{k|k}$ according to the following equation: $P_{k|k}=(I-K_{k}H_{k})P_{k|k-1}{\left(I-K_{k}H_{k}\right)}^{T}+K_{k}{\hat{C}}_{k}K_{k}^{T}$ (27). This formulation helps ensure the positive definiteness and numerical stability of the matrix.

Step 9: Iteration Loop

Take the updated state estimate ${\hat{x}}_{k|k}$ and error covariance matrix $P_{k|k}$ obtained in this iteration as the initial values for the filtering process at the next time step (k+1). Return to Step 2 and repeat the prediction and update processes to achieve continuous, adaptive state estimation throughout the entire system operation.

This process, by dynamically adjusting the innovation covariance estimation and the Kalman gain, enables the AEKF algorithm to maintain high-precision and strongly robust state estimation performance even in the presence of system model uncertainties or external disturbances.

## 4. Simulation Verification and Discussion

The proposed AEKF algorithm incorporates a robust weighting mechanism based on the Mahalanobis distance and a dynamic forgetting factor, which collectively enhance the filter’s adaptability to system uncertainties. Theoretically, this adaptive mechanism mitigates model mismatch issues induced by parameter variations through real-time adjustment of the confidence assigned to measurement innovations. When motor parameters such as stator resistance or inductance deviate from their nominal values—due to thermal effects or operational wear—the conventional EKF often exhibits biased state estimates and sluggish convergence. In contrast, the AEKF continuously updates its internal innovation covariance estimate, enabling the filter to recalibrate its gain dynamically. This process reduces reliance on pre-defined noise statistics and fixed model parameters, thereby alleviating performance degradation under parameter variations. The following simulations validate that this adaptive structure effectively sustains estimation accuracy and robustness in the presence of model mismatches.

**Figure 2:**
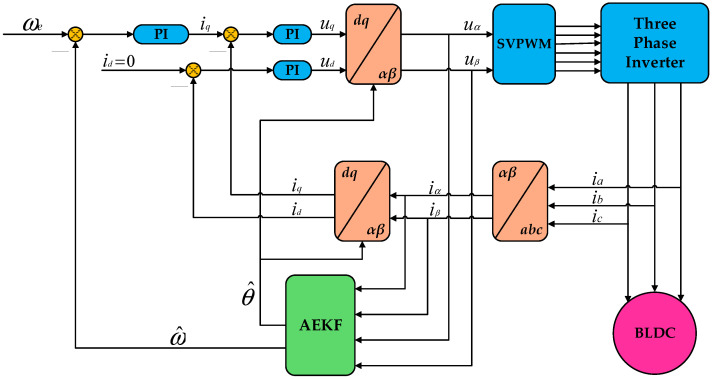
Simulation schematic of the BLDC control system.

A BLDC control model was established in Matlab/Simulink with a simulation time set to 8 s. The simulation schematic diagram of the BLDC control system is shown in Figure 2, which includes modules such as PI control, Clarke transformation, Park transformation, a standard Space Vector Pulse Width Modulation (SVPWM) algorithm [32], a three-phase inverter circuit, and the AEKF.

Considering the widespread demand for high-efficiency, high-dynamic-response control systems for brushless DC motors in fields such as industrial automation, precision instrumentation, and consumer electronics, this paper selects a low-power BLDC as the subject for simulation research. This type of motor is frequently operated under conditions involving frequent start-stop cycles, variable-speed operation, or load disturbances in applications such as medical devices, precision optical equipment, and actuators for automated instrumentation, imposing high requirements on the accuracy of rotor position observation and the system’s disturbance rejection capability. The specific parameters of the motor selected for the experiment are as follows: rated voltage 24 V, rated power 80 W, rated speed 2000 rpm, rated torque 0.4 N·m, number of pole pairs 3, stator resistance 15 mΩ, and stator inductance 0.2 mH, as shown in Table 1.

A BLDC control model was constructed, based on which the conventional EKF algorithm and the improved AEKF algorithm were respectively applied under identical operating conditions. To ensure a fair comparison, the fixed noise covariance matrices employed in the conventional EKF were set identically to the corresponding initial matrices used in the proposed AEKF. Specifically, the process noise covariance, measurement noise covariance, and the initial innovation covariance for the AEKF were all initialized with the same values adopted in the conventional EKF. Furthermore, all other shared parameters—including the PI controller gains and the sampling period—were kept consistent between the two algorithms. This configuration ensures that any performance differences observed in the comparative experiments are solely attributable to the algorithmic enhancements. Comparative experiments were conducted on motor rotor position during the low-speed startup phase, speed step-change tests at different speeds, and sudden load application tests at different speeds. The resulting curves depicting motor rotor position and speed changes were obtained from the simulation results.

To evaluate the performance of the proposed Adaptive Extended Kalman Filter (AEKF) algorithm during the low-speed startup phase, where the back-EMF signal is weak and model uncertainty is significant, a set of comparative simulation experiments was designed. The experiment focused on a sensorless BLDC, accelerating it from standstill to 400 rpm under no-load conditions. Real-time rotor position estimation was performed using both the traditional EKF and the proposed AEKF algorithm. The experiment specifically focused on the tracking accuracy of the rotor’s actual motion trajectory, the magnitude of the initial lag, and the dynamic convergence characteristics of the two algorithms under conditions of low signal-to-noise ratio and strong nonlinearity. In the simulation, the motor parameters all adopted the actual typical values listed in Table 1, and parameters related to algorithm implementation, such as the control cycle and initial noise covariance values, were kept consistent to ensure a fair comparison. By recording and comparing the estimated and actual position curves, the aim was to quantitatively analyze the effectiveness of the AEKF algorithm in enhancing observation robustness and dynamic response speed in the low-speed region.

**Figure 3:**
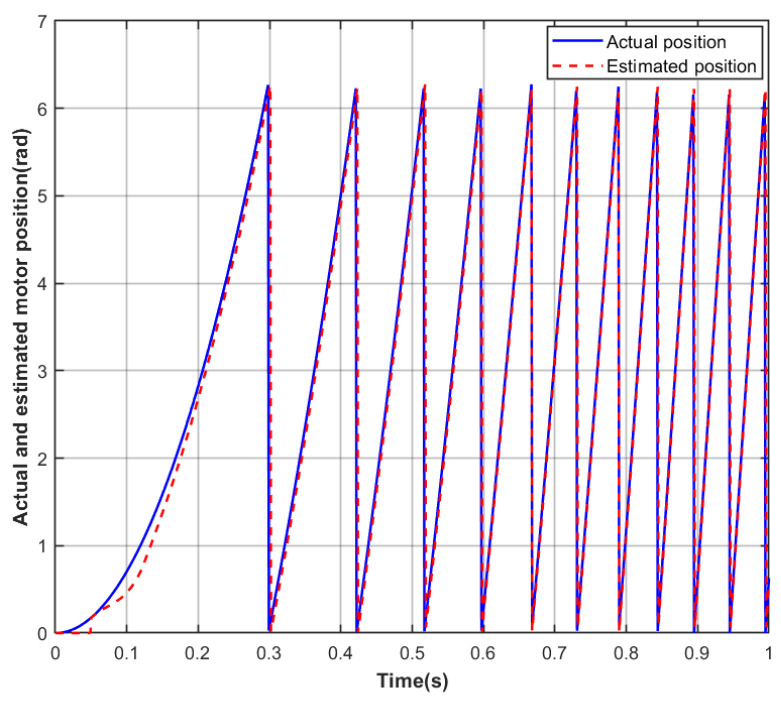
EKF-estimated versus actual rotor position during startup.

As shown in Figure 3, under no-load motor operation, during the startup transient from 0 rpm to 400 rpm, a significant deviation exists between the rotor position estimation curve obtained using the EKF algorithm and the actual position curve. In the low-speed startup phase, this algorithm’s observation performance reveals notable deficiencies: at the beginning of acceleration, the estimated rotor position fails to promptly follow the actual rotor motion, exhibiting a brief period of tracking stagnation. Subsequently, although the estimate begins to respond, its dynamic tracking process exhibits a delay, causing the estimation error to increase within a very short time, with a lag of approximately 0.31 rad. This result indicates that under low-speed conditions with low back-EMF amplitude and poor signal-to-noise ratio, the traditional EKF, based on fixed noise covariance and a linearized model, struggles to effectively handle the system’s strong nonlinearity and model uncertainty. This leads to issues such as slow dynamic response of the observer and significantly increased convergence lag, which may consequently affect the precision and dynamic performance of the motor’s vector control.

**Figure 4:**
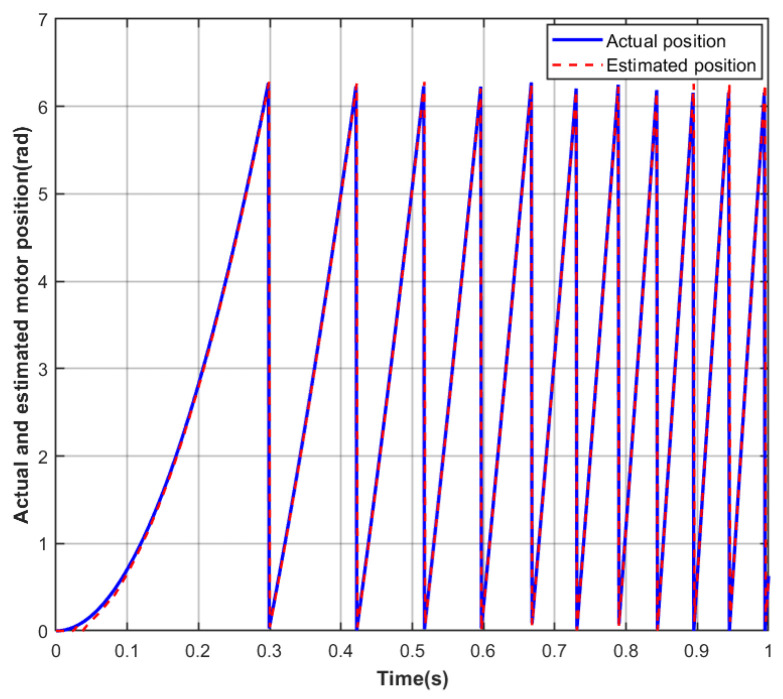
AEKF-estimated versus actual rotor position during startup.

As shown in Figure 4, under no-load motor operation during the acceleration startup from 0 rpm to 400 rpm, the rotor position estimation results obtained using the AEKF algorithm are presented. In the low-speed startup phase, characterized by low back-EMF signal amplitude and significant model uncertainty, the algorithm demonstrates excellent dynamic observation performance. The observer output closely follows the actual rotor motion from the initial moment, with the initial tracking lag effectively suppressed to a relatively low level of approximately 0.24 rad. Furthermore, compared to the traditional EKF results shown in Figure 3, the convergence rate of the AEKF estimation error is significantly faster, exhibiting smooth and rapid decay characteristics. This performance improvement primarily benefits from the adaptive estimation capability of the AEKF algorithm, which enables online real-time adjustment of the statistical properties of process and measurement noise, thereby effectively overcoming the model mismatch problem faced by fixed-parameter filters during highly dynamic operation and changing conditions. The simulation results indicate that the AEKF algorithm significantly enhances the rotor position observation accuracy and dynamic response speed of the sensorless motor in the low-speed region.

To further verify the tracking performance and robustness of the proposed AEKF algorithm under conditions of speed dynamic variation, a speed step-change experiment was designed. This experiment aims to investigate key dynamic indicators of the algorithm during abrupt speed changes, including tracking speed of the target speed, overshoot suppression capability, and steady-state accuracy. The simulation experiment scheme involves setting the motor under no-load conditions to accelerate from standstill to speed ranges of 400 rpm and 800 rpm, as well as 600 rpm and 1200 rpm, respectively, with stepwise speed increases and decreases within each speed range. During the experiment, parameters such as the controller’s proportional-integral gains, sampling period, and initial noise covariance values were kept consistent with those used for the traditional EKF, to highlight the performance differences attributable solely to the algorithm improvement. By recording and comparing the speed response curves of both algorithms under different speed steps, with a focus on quantitative metrics such as settling time, maximum overshoot, and steady-state error, a systematic evaluation of the AEKF’s superiority in handling system dynamic changes was conducted.

**Figure 5:**
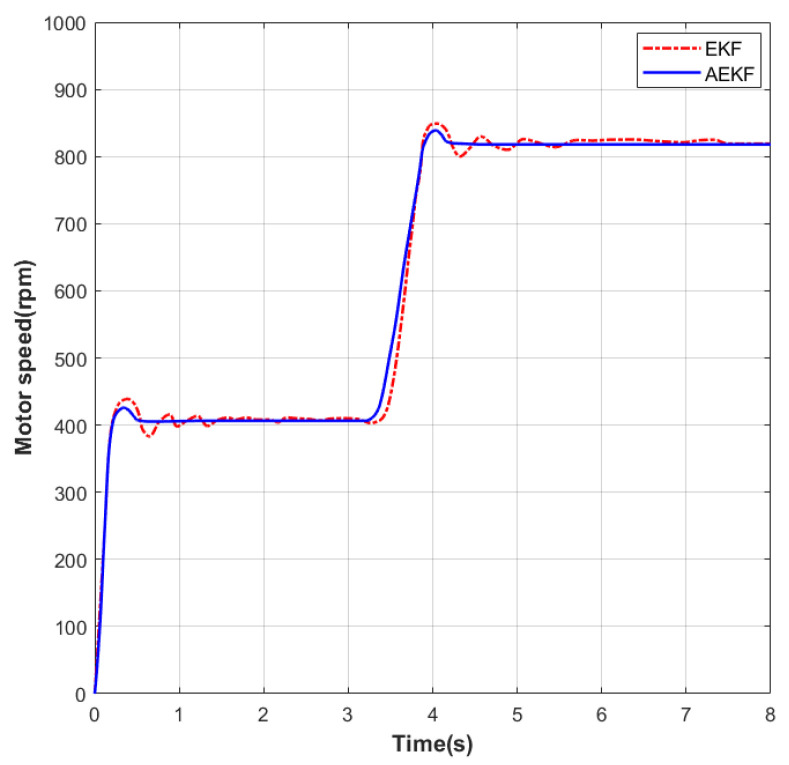
Speed profile during acceleration test from 400 to 800 rpm.

As shown in Figure 5, a speed step-change experiment was performed on the motor under no-load conditions. The initial speed was set to 0 rpm. In the first phase, the speed was increased to 400 rpm. Under EKF control, the settling time was 0.89 s and the maximum overshoot was 26 rpm; under the improved AEKF control, the settling time was 0.81 s and the maximum overshoot was 22 rpm. During the steady-state phase at this speed, the maximum static error under EKF was 7 rpm, while under AEKF it was 5 rpm. In the second phase, the speed was increased from 400 rpm to 800 rpm. Under EKF control, the settling time was 1.15 s and the maximum overshoot was 35 rpm; under AEKF control, the settling time was 1.03 s and the maximum overshoot was 29 rpm. During the steady-state phase at this speed, the maximum static error under EKF was 17 rpm, while under AEKF it was 14 rpm.

It should be noted that the reduction in steady-state speed error is directly attributed to the more accurate and less noisy speed estimate provided by the AEKF, which improves the input signal quality for the speed-loop PI controller, rather than any alteration to the controller’s own parameters or structure.

**Figure 6:**
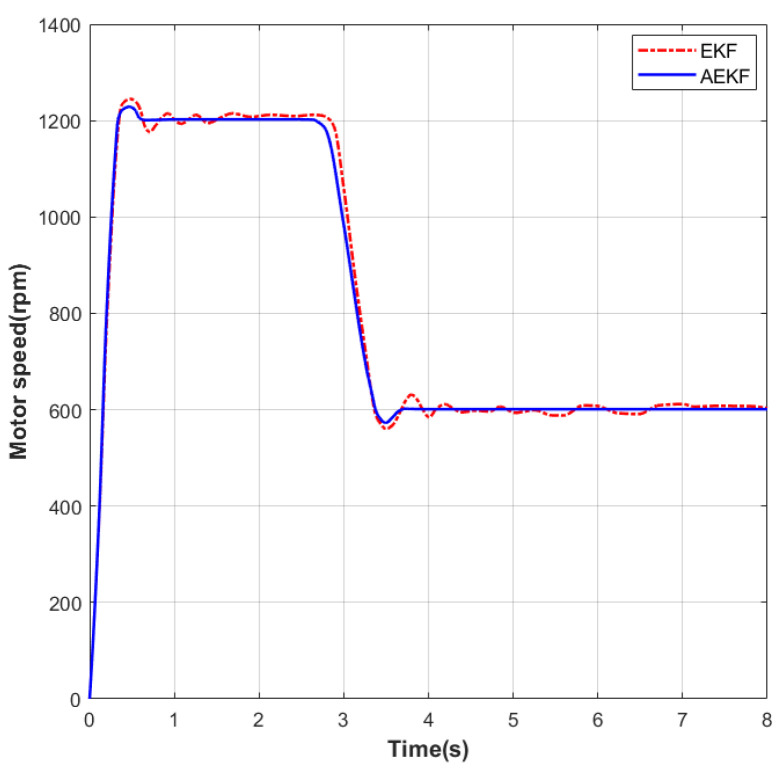
Speed profile during deceleration test from 1200 to 600 rpm.

As shown in Figure 6, a speed step-change experiment was conducted on the motor under no-load conditions. The initial speed was set to 0 rpm. In the first phase, the speed was increased to 1200 rpm. Under EKF control, the settling time was 1.30 s and the maximum overshoot was 43 rpm; under the improved AEKF control, the settling time was 1.16 s and the maximum overshoot was 37 rpm. During the steady-state phase at this speed, the maximum static error under EKF was 24 rpm, while under AEKF it was 20 rpm. In the second phase, the speed was decelerated from 1200 rpm to 600 rpm. Under EKF control, the settling time was 1.20 s and the maximum overshoot was 33 rpm; under AEKF control, the settling time was 1.08 s and the maximum overshoot was 26 rpm. During the steady-state phase at this speed, the maximum static error under EKF was 14 rpm, while under AEKF it was 11 rpm.

To systematically evaluate the dynamic disturbance rejection capability and robustness of the proposed AEKF algorithm under external disturbances, this paper further conducted sudden load application experiments. These experiments aim to simulate common load step changes in practical applications, investigating the algorithm’s performance in maintaining speed stability, suppressing dynamic fluctuations, and quickly recovering to steady-state under abrupt torque changes. The experiment selected two typical speed points, 800 rpm and 1200 rpm. After the motor started under no-load and reached steady state, a load torque of 0.1 N·m was suddenly applied at the 3 s mark. In the experiment, parameters such as the PI controller gains, sampling period, and initial noise covariance values were kept consistent with those used for the traditional EKF to ensure a fair comparison. By recording and analyzing the speed response curves of both algorithms before and after the load transient, with a focus on dynamic metrics such as settling time, maximum overshoot, and steady-state error, a comprehensive verification of the AEKF’s superior adaptability and robust performance in responding to sudden external disturbances was conducted.

**Figure 7:**
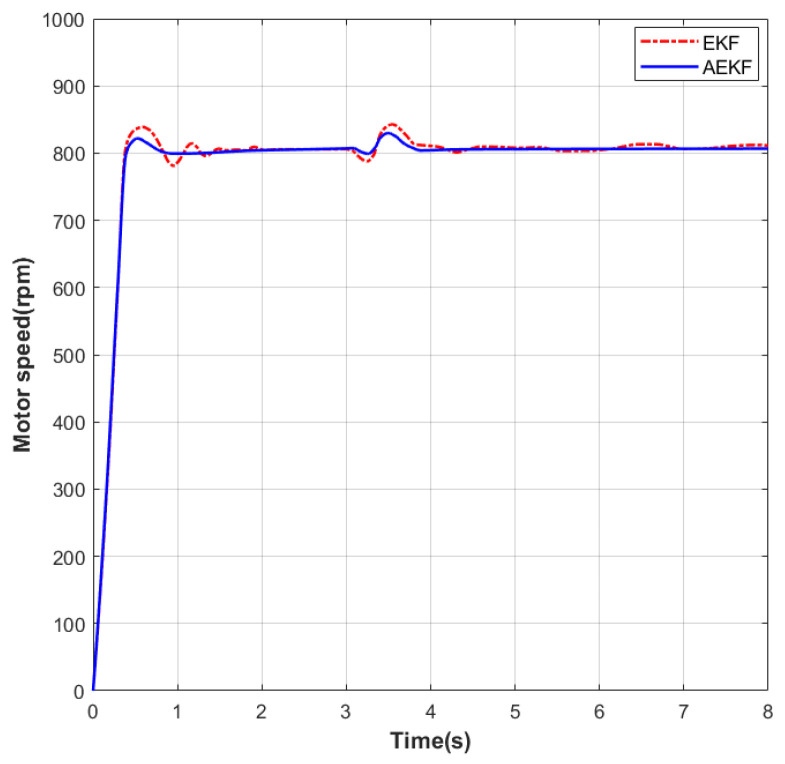
Dynamic speed response at 800 rpm following a sudden load application.

As shown in Figure 7, a sudden load application experiment was performed on the motor under no-load conditions. The initial speed was set to 0 rpm. In the first phase, the speed was increased to 800 rpm. Under EKF control, the settling time was 1.18 s and the maximum overshoot was 35 rpm; under the improved AEKF control, the settling time was 1.07 s and the maximum overshoot was 28 rpm. During the steady-state phase at this speed, the maximum static error under EKF was 18 rpm, while under AEKF it was 14 rpm. In the second phase, at the 3 s mark, a sudden load of 0.1 N·m was applied to the motor. Subsequently, under EKF control, the settling time was 1.33 s and the maximum overshoot was 39 rpm; under AEKF control, the settling time was 1.18 s and the maximum overshoot was 32 rpm. During the steady-state phase at this speed, the maximum static error under EKF was 18 rpm, while under AEKF it was 13 rpm.

**Figure 8:**
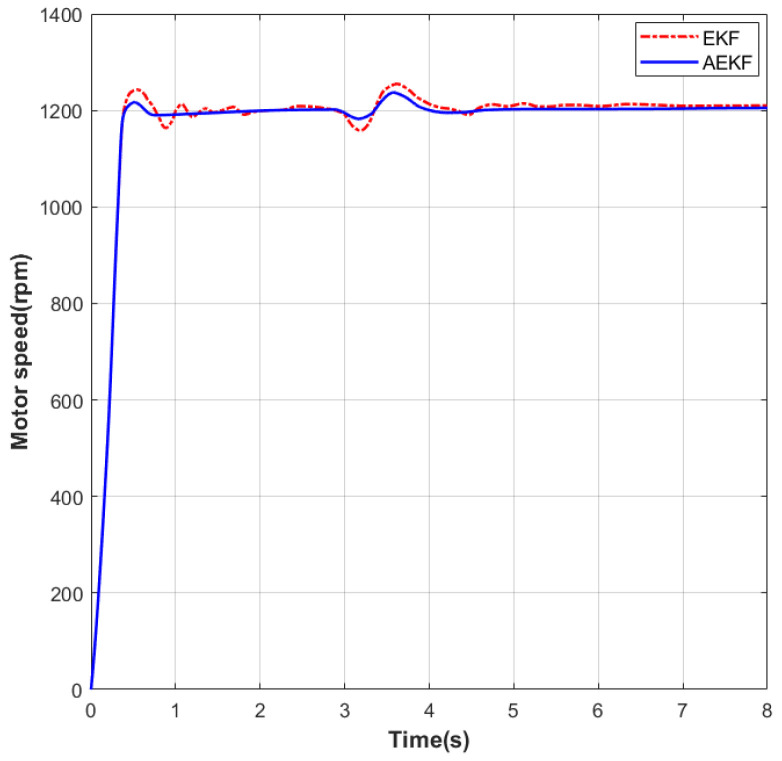
Dynamic Speed Response at 1200 rpm Following a Sudden Load Application.

As shown in Figure 8, a sudden load application experiment was conducted on the motor under no-load conditions. The initial speed was set to 0 rpm. In the first phase, the speed was increased to 1200 rpm. Under EKF control, the settling time was 1.35 s and the maximum overshoot was 44 rpm; under the improved AEKF control, the settling time was 1.21 s and the maximum overshoot was 36 rpm. During the steady-state phase at this speed, the maximum static error under EKF was 25 rpm, while under AEKF it was 21 rpm. In the second phase, at the 3 s mark, a sudden load of 0.1 N·m was applied to the motor. Subsequently, under EKF control, the settling time was 1.55 s and the maximum overshoot was 48 rpm; under AEKF control, the settling time was 1.25 s and the maximum overshoot was 38 rpm. During the steady-state phase at this speed, the maximum static error under EKF was 26 rpm, while under AEKF it was 20 rpm.

Based on the comparative analysis of the aforementioned simulation results, during the low-speed startup phase, the AEKF effectively reduced the initial tracking lag and convergence time in rotor position estimation, demonstrating stronger adaptability to low back-EMF operating conditions. Throughout dynamic processes involving speed step changes and sudden load applications, the algorithm also exhibited faster settling speed, lower overshoot, and smaller steady-state errors, reflecting its excellent dynamic tracking capability and disturbance rejection robustness.

## 5. Conclusions

This paper addresses the challenges of model mismatch and estimation performance degradation in sensorless brushless DC motor (BLDC) control under dynamic transients and complex disturbances by designing an Adaptive Extended Kalman Filter (AEKF) algorithm. Building upon the traditional EKF framework, the proposed algorithm introduces a robust weighting adjustment mechanism based on the Mahalanobis distance and a dynamically adaptive forgetting factor. This constructs an estimation structure capable of online innovation covariance updating, thereby significantly enhancing the state observer’s adaptability to system uncertainties and external disturbances.

The simulation results demonstrate that the designed AEKF algorithm exhibits superior overall performance compared to the traditional EKF under various typical operating conditions, including low-speed start-up, speed step changes, and sudden load applications. Specifically, during low-speed start-up, the AEKF reduces the initial tracking lag in rotor position estimation by approximately 22.6%. In the speed step-change experiments, the settling time is shortened by an average of about 11.3%, the maximum overshoot is reduced by an average of about 15.4%, and the steady-state error is decreased by an average of about 20.6%. Under sudden load conditions, the settling time is shortened by an average of about 14.1%, the overshoot is reduced by an average of about 17.9%, and the steady-state error is lowered by an average of about 22.2%. These quantitative results consistently indicate that the AEKF possesses faster convergence speed, smaller tracking lag, and better steady-state accuracy during dynamic processes, while also significantly enhancing the system’s adaptability and robustness to external disturbances and model uncertainties. The findings validate the effectiveness of this algorithm in improving the dynamic response precision and operational stability of sensorless BLDC control systems. Furthermore, the proposed Adaptive Extended Kalman Filter algorithm demonstrates the potential to significantly lower the minimum speed required for reliable sensorless estimation. The experimental results presented in Section 4 show that the algorithm maintains excellent estimation accuracy and dynamic performance even at 400 rpm, where the back-EMF signal amplitude is substantially attenuated. Based on these findings and the algorithm’s ability to effectively suppress common low-speed issues such as model mismatch and noise sensitivity, it can be inferred that the AEKF is capable of delivering reliable estimation performance down to approximately 200 rpm. This represents a meaningful extension of the stable sensorless operating range and provides an effective solution to a key challenge in low-speed and start-up control applications.

This research not only provides an adaptive and robust filtering solution for state observation in high-dynamic-performance motor drive systems but also offers theoretical and technical references for further exploration of intelligent estimation methods for nonlinear, strongly coupled electromechanical systems. Future work will systematically conduct parameter perturbation simulations and experiments to quantitatively evaluate the robustness boundary of the algorithm under varying motor parameters and operating conditions. Furthermore, implementation on real-time hardware platforms will be pursued to validate its practical feasibility in embedded systems.

Furthermore, integrating the adaptive observer proposed in this study with advanced intelligent fault diagnosis techniques represents a significant direction for building highly reliable motor drive systems [33]. For instance, lightweight diagnosis methods based on multi-source information data-layer fusion can utilize the high-precision state estimates (such as speed and current) provided by this observer as key input features. This enables online monitoring and diagnosis of incipient faults in motor components like windings, bearings, and power circuits. The deep integration of control and health management facilitates the development of next-generation drive systems with predictive maintenance capabilities.

## Figures and Tables

**Table 1:** Motor parameter specifications.

Parameter	Value	Unit
Rated voltage	24	V
Rated power	80	W
Rated speed	2000	rpm
Rated torque	0.4	N·m
Number of pole pairs	3	/
Stator resistance	15	mΩ
Stator inductance	0.2	mH

## Data Availability

The datasets generated and analyzed during this study are not publicly available due to being part of an ongoing larger study. However, they are available from the corresponding author on reasonable request.

## Abbreviations

The following abbreviations are used in this manuscript:

BLDC	Brushless DC Motor
EKF	Extended Kalman Filter
AEKF	Adaptive Extended Kalman Filter
SMO	Sliding Mode Observer
MRAS	Model Reference Adaptive System
back-EMF	Back Electromotive Force
PLL	Phase-Locked Loop
PI	Proportional-Integral
SVPWM	Space Vector Pulse Width Modulation
